# High-Resolution Eye-Tracking System for Accurate Measurement of Short-Latency Ocular Following Responses: Development and Observational Study

**DOI:** 10.2196/64353

**Published:** 2024-12-09

**Authors:** Aleksandar Miladinović, Christian Quaia, Simone Kresevic, Miloš Ajčević, Laura Diplotti, Paola Michieletto, Agostino Accardo, Stefano Pensiero

**Affiliations:** 1 Institute for Maternal and Child Health-IRCCS, Trieste, 34100, Italy, 39 0405587124; 2 Laboratory of Sensorimotor Research, National Eye Institute, National Institutes of Health, Department of Health and Human Services, Bethesda, MD, United States; 3 Department of Engineering and Architecture, University of Trieste, Trieste, Italy

**Keywords:** ocular following response, stereopsis, video-oculography, ocular, tracker, vision, pediatric, children, youth, infrared, algorithm, eye tracking

## Abstract

**Background:**

Ocular following responses (OFRs)—small-amplitude, short-latency reflexive eye movements—have been used to study visual motion processing, with potential diagnostic applications. However, they are difficult to record with commercial, video-based eye trackers, especially in children.

**Objective:**

We aimed to design and develop a noninvasive eye tracker specialized for measuring OFRs, trading off lower temporal resolution and a smaller range for higher spatial resolution.

**Methods:**

We developed a high-resolution eye-tracking system based on a high-resolution camera operating in the near-infrared spectral range, coupled with infrared illuminators and a dedicated postprocessing pipeline, optimized to measure OFRs in children. To assess its performance, we: (1) evaluated our algorithm for compensating small head movements in both artificial and real-world settings, (2) compared OFRs measured simultaneously by our system and a reference scleral search coil eye-tracking system, and (3) tested the system’s ability to measure OFRs in a clinical setting with children.

**Results:**

The simultaneous measurement by our system and a reference system showed that our system achieved an in vivo resolution of approximately 0.06°, which is sufficient for recording OFRs. Head motion compensation was successfully tested, showing a displacement error of less than 5 μm. Finally, robust OFRs were detected in 16 children during recording sessions lasting less than 5 minutes.

**Conclusions:**

Our high-resolution, noninvasive eye-tracking system successfully detected OFRs with minimal need for subject cooperation. The system effectively addresses the limits of other OFR measurement methods and offers a versatile solution suitable for clinical applications, particularly in children, where eye tracking is more challenging. The system could potentially be suitable for diagnostic applications, particularly in pediatric populations where early detection of visual disorders like stereodeficiencies is critical.

## Introduction

Eye movements have long been used to gain insights into the operation of brain structures involved in motor and cognitive processes. Furthermore, they provide opportunities to diagnose nervous system pathologies through their oculomotor signatures [1], including parkinsonian syndromes [2,3], amyotrophic lateral sclerosis [4], Huntington disease [5], Alzheimer disease [6], and minimal hepatic encephalopathy [7].

In our daily lives, we unconsciously engage in various types of eye movements. The involuntary eye movements that are responsible for maintaining the stability of images on the retina include the vestibulo-ocular reflex, smooth pursuit, and optokinetic nystagmus. Abrupt onset of visual motion induces reflexive eye movements with ultra-short latencies, known as ocular following responses (OFRs). These responses constitute the initial component of the optokinetic nystagmus response, supporting the translational vestibulo-ocular reflex in gaze stabilization [8]. Despite their small magnitude, typically compensating for only 4%‐10% of retinal slip, OFRs have proven valuable for investigating the processes underlying visual motion processing in humans and nonhuman primates [9-20]. Furthermore, by using scleral search coils [11] to record eye movements in stereoblind adults, their potential for diagnosing stereoanomalies has been recently revealed [21].

Stereoblindness, the inability to use the disparity between the retinal images from the 2 eyes to sense depth, is an often irreversible central nervous system disorder, with a prevalence of around 7% [22]. It is usually associated with, and often a result of, strabismus (misalignment of the 2 eyes) or anisometropia (a large difference in the refractive power between the 2 eyes). If not treated early, it often leads to amblyopia, a central (and thus not correctable with lenses) visual acuity deficit in 1 eye.

Early diagnosis during the critical childhood period of visual development is crucial to prevent the development of amblyopia, with early intervention (ideally, during the first 12‐36 months of life) leading to improved outcomes [23-25]. Several clinical tests are currently available to assess binocular depth perception, such as the Titmus, TNO, and Lang tests, but they require patient cooperation, and so are poorly suited to assess stereodeficiencies in infants and young children [26], which remains a challenge.

Measuring OFRs requires minimal subject cooperation. As they are mediated by disparity-sensitive cortical neurons, sensitive to interocular correlations [27], they are affected by stereoblindness [27]. Accordingly, they have the potential to be used to detect stereo deficits in an objective manner, particularly in patient populations not well suited to current methods (such as children, especially preverbal ones, and nonverbal individuals of any age). Despite their potential, due to their small amplitude (usually less than half a degree), OFRs are rarely recorded. Typically, they have been measured using costly eye-tracking equipment, such as scleral search coils and dual-Purkinje eye trackers, which is almost never available in clinical practice. Recently, noninvasive, general-purpose, commercial, video-based eye-tracking systems have also been used to record OFRs, but these require averaging responses over many, often 100 [28-30] or more (up to 400 [30]), repeated presentations of the same stimulus, collected over multiple sessions, a process unsuitable for clinical practice and in pediatric populations.

This need for averaging is due to the limited spatial resolution of commercial eye trackers, which in turn is a consequence of their need to provide high temporal resolution and operate over a large range of eye positions. However, this is the wrong trade-off for the low speed and small size of OFRs, and a video system operating with a different trade-off might achieve the spatial accuracy needed to reliably record OFRs. The aim of our study is to conceptualize, design, and implement a high-resolution eye tracking system, with compensation for small head movements, suitable for noninvasive measurement of OFRs in children. This system is intended to address the challenges associated with traditional eye tracking methods, maximizing subject comfort and minimizing recording duration and required subject cooperation, making it suitable for application in pediatric clinical contexts.

## Methods

### Ethical Considerations

Each participant’s parent or legal guardian provided written informed consent prior to their child’s involvement in the study. They were informed that the test would not directly benefit their child, that participation was entirely voluntary and unrelated to any clinical care the child was receiving at the eye clinic that day, and that their decision to decline participation would not impact their child’s clinical care at the hospital, either currently or in the future. The equipment used in the study was demonstrated to the parent, and a few sample trials were conducted to illustrate the visual stimulation and what was expected of the child. Additionally, the parent was present throughout the testing session. No compensation was provided to participants in this study. To ensure privacy, all data were anonymized prior to analysis to prevent any potential identification of participants. The study (RC 31/24) was conducted in accordance with the Declaration of Helsinki and was approved by the Institutional Scientific Board of the Istituto di Ricovero e Cura a Carattere Scientifico (IRCCS) “Burlo Garofolo.”

### Requirements

**Figure 1.**
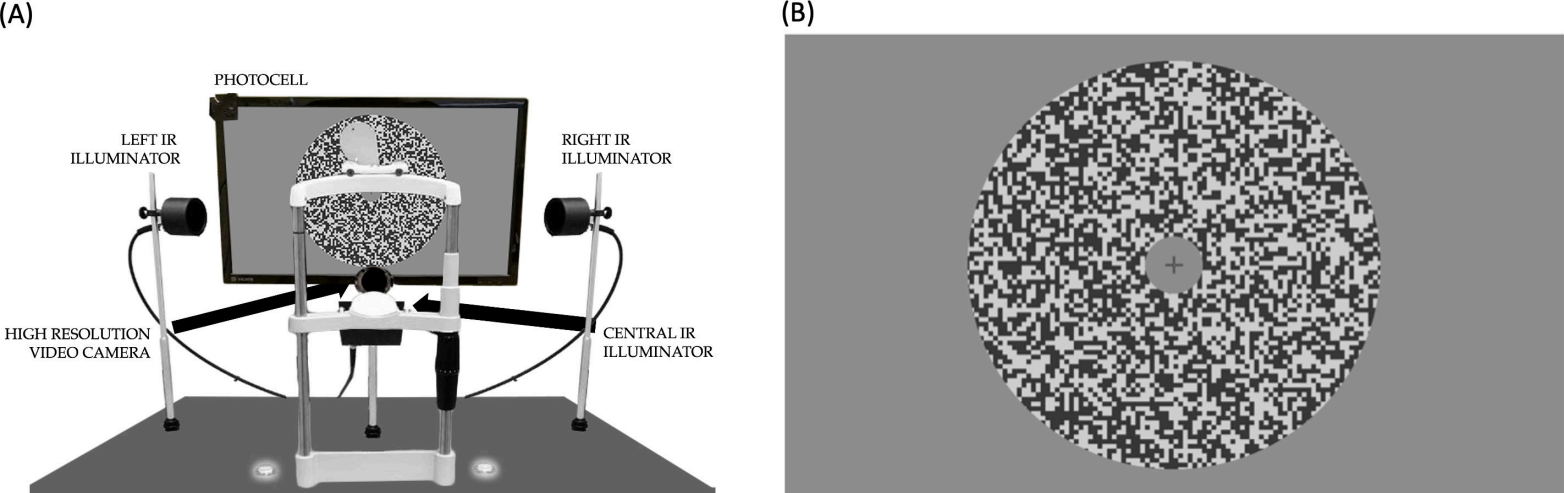
**(A**) Our custom-designed eye-tracking apparatus. (**B**) Sample random-dot stimulus used to induce ocular following responses in experiments. IR: infrared.

OFRs are reflexive movements, with consistent temporal dynamics and a latency (for high-contrast stimuli) of 70‐80 milliseconds in humans [28,31]. In a typical experiment, 4 or more different conditions, each corresponding to a different motion stimulus, are presented in succession. Each presentation represents a trial, and each condition is presented once in a block of trials. Multiple such blocks are recorded (possibly over multiple sessions) so that the average response to each condition can then be computed. Typically, a trial lasts between 1.5 and 2 seconds, with a fixation period of approximately 1 second, during which the first frame of the stimulus and a fixation cross are shown on the screen (Figure 1B). This is followed by a short (usually 200-millisecond) period of stimulus motion (during which the fixation cross is not visible), followed by a short period during which a blank screen is presented. Subjects are usually encouraged to limit eye blinking to this blank period.

In some cases, the latency is itself a focus of the study; therefore, high temporal resolution is necessary. However, in most cases, the measure of interest is the magnitude of the response (ie, the displacement of the eyes) in the so-called open-loop period [10]. This is the period during which the eye movement itself does not alter the projection of the visual input on the retina, and it goes from the movement latency to twice the latency (after this time, the retinal velocity of the stimulus would become equal to the difference between the actual velocity of the stimulus and the velocity of the eye 1 movement latency earlier). The onset latency varies somewhat from subject to subject and as a function of the contrast and size of the stimulus [10,13,15], but for high-contrast large stimuli, it varies between 70 and 80 milliseconds. Under such conditions, quantifying the OFR as the eye displacement between 80 milliseconds and 160 milliseconds after stimulus onset is appropriate for most subjects.

The most basic system for measuring OFRs must then determine, with the highest resolution possible, the displacement of 1 eye during the open-loop period. The stability of eye fixation during the period that precedes the onset of the OFRs should also be verified, discarding trials in which the subject moved their eyes around stimulus onset (such motion would alter the motion of the stimulus on the retina during the open-loop period). The acquisition of just 3 frames (motion onset, 80 milliseconds later, and 80 milliseconds after that) should suffice for measuring at least OFRs to the simplest stimuli [32]. As we are interested in the motion of the eye in the head, it is important to accurately estimate and subtract off movements of the head over the same time period. More complex visual stimulations, in which the dynamics or the latency of OFRs might be informative, would require higher temporal resolutions. In those cases, the high temporal resolution of an eye coil system (1 millisecond) would certainly be helpful, but it is hard to envision situations in which acquiring more than 10 frames over a 200-millisecond period would be strictly required to answer a scientific or clinical question. Once the images are acquired by the camera, there would then be between 1 and 2 seconds, depending on the exact trial duration, during which the images could be downloaded to disk for offline analysis.

Given the typical size of OFRs, with a maximum displacement during the open-loop period ranging across subjects between 0.05° and 0.5°, a resolution of less than 0.05° would be highly desirable. To put this in perspective, peak-to-peak noise levels with a scleral search coil system, the most sensitive system for recording eye movements, are typically less than 0.03° [33]. In contrast, commercial eye trackers often have difficulties in reliably detecting eye displacements smaller than 0.5° [34,35]. With an average eye diameter of 22‐24 mm, 0.05° corresponds to a pupil displacement of approximately 10 µm. Head movements of such small amplitudes must therefore also be reliably detected.

We designed and built a video-oculography system, utilizing a combination of off-the-shelf components and custom-designed elements, to fulfill these specifications.

### Video Acquisition System

The image acquisition system we designed consists of a high-resolution camera, infrared (IR) illuminators, a photocell to be mounted to the monitor, and an Arduino controller to monitor the output of the photocell and trigger a frame acquisition from the camera. The camera we selected is a monochromatic FLIR Grasshopper 3 GS3-U3-51S5M-C [36]. It contains a Sony IMX250 ⅔” complementary metal-oxide-semiconductor sensor, with a resolution of 2448 × 2048 pixels (approximately 5 megapixels), square pixels with a side of 3.45 µm, and a maximum acquisition rate (at full frame size) of 75 Hz. We paired it with a Computar M5028-MPW2 C-Mount ⅔” 50 mm, f/2.8 lens [37]. Since we are interested in recording in the near-IR spectral range, we blocked the visible spectrum by placing in front of the lens a Hoya R72 IR filter with a cutoff wavelength of 720 nm [38]. To ensure proper lighting, we used 3 IR LED illuminators (1 on each side of the subject and 1 in front of and below the subject). We assembled them using multiple (2 for the front, 12 for the sides) 800-nm LEDs held in custom-designed 3D-printed enclosures. A common problem with commercial eye trackers is that the sustained illumination required by high temporal resolution often results in eye dryness and subject discomfort. To allow for bright IR illumination of the eye during image acquisition while minimizing the amount of IR power delivered to the eye, our illuminators are pulsed for only 2 milliseconds at a time, synchronized with the acquisition of a frame by the camera (the camera exposure duration was set to 1 millisecond to minimize motion blur). When recording 3 frames/trial, in an experiment with 4 stimulus conditions (the bare minimum for OFR experiments), 30 repeated presentations of each condition, and a 2-second trial duration, this results in a 4-minute recording experiment during which the illuminators are lit for only 720 milliseconds. Additional illumination occurs in the setup period required to properly focus the lens on the subject’s eye. With an experienced operator, this process typically lasts less than 30 seconds; during this process, frames are acquired at 10 Hz (and the illuminators are lit 2 milliseconds every 100 miliseconds), resulting in an additional 600 milliseconds of IR illumination.

To control the timing of the camera shutter and the illuminators, we designed and programmed an Arduino-based controller and connected a photocell placed in front of the top left corner of the monitor to an Arduino analog-to-digital input. Our stimuli are then designed so that the luminance of the area of the screen under the photocell increases on the frame on which the fixation point is turned off and the experimental stimulus starts drifting. The Arduino-controlled circuit detects this change and sends a 2-millisecond transistor-transistor logic (TTL) pulse to the analog circuit that powers the IR illuminators and the camera shutter. The illuminators are turned on essentially instantly, whereas the camera, which is configured to trigger on the rising edge of the TTL pulse, begins the acquisition of a frame approximately 0.3 milliseconds later. Shutter aperture is set to last 1 millisecond, irrespective of the duration of the TTL pulse. The acquisition of successive frames (with associated illumination pulses) is then triggered automatically by the Arduino controller at the desired delays (with submillisecond resolution). The controller we designed allows for the manual selection (using a rotating knob) of 1 of 9 programs, each associated with different numbers and timings of acquisition frames (relative to the first one, which is always triggered by the photocell). The timing of these sequences can be easily customized by modifying the Arduino code. As mentioned previously, the camera we selected has a pixel size of 3.45 µm, and the minimum focusing distance of the lens we selected is approximately 50 cm (appropriate also for the distance between the subject and the monitor, allowing us to place the camera under the monitor). A camera pixel will therefore cover a square with a side of approximately 40 µm on the subject’s eye. Given our desired 10 µm resolution (as detailed previously), our image analysis pipeline will need to detect displacements of one-fourth of a pixel or better. Since even very small translations or vibrations of the head would introduce artifacts of the same order of magnitude as the movements we are attempting to measure, these need to be compensated for.

### Head Movement Compensation

The problem of head stabilization is common to all video-based systems (whereas scleral search coil systems are insensitive to small head translations). Gross head stabilization is usually provided by using a chin and forehead rest, often augmented with a headband. This is sufficient for situations in which only a coarse localization of the eye is required, but it is not sufficient when accurate determination of eye position is required.

One solution to the problem is to tightly stabilize the head, which is usually accomplished either through a bite bar (in which the subject’s teeth are trapped in a dental mold fixed to the recording apparatus) or through a tight-fitting helmet that is then mechanically or magnetically held firmly in place. However, both of these solutions are impractical, uncomfortable for the subject, and ill-suited to pediatric clinical environments.

A noninvasive solution to this problem, used by most commercial video eye trackers, is to track the reflex on the cornea caused by an IR LED (the corneal reflex or first Purkinje image) [39,40]. When the eye rotates in the head, this reflex does not (to a first-order approximation) move in the image plane (whereas the pupil’s center does). In contrast, when the head translates, the reflex translates with it (and so does the pupil’s center). To estimate from video images the motion of the eye when the head is not perfectly stabilized, one can then subtract the displacement of the corneal reflex (an estimate of head-in-space motion) from the displacement of the pupil center (an estimate of eye-in-head + head-in-space motion). The resolution of the final measure is limited by the resolution with which the displacement of the corneal reflex can be tracked; in our case, this will also have to be in the order of one-fourth of a pixel or better. We attempted to use this approach but found it unsatisfactory for two reasons: (1) properly placing the illuminators to get an appropriate corneal reflex can be cumbersome and time-consuming, and (2) we could not reliably achieve the desired resolution given the small area covered by the corneal reflex [35].

To more accurately track small movements of the head, we placed an IR-absorbing black circle on a small sticker (which we call a “head marker”) just under the bridge of the nose of the subject. We chose this location because it is close to the pupil we image (allowing them to both fit in the same photo frame) and because this area of the face is minimally sensitive to changes in facial expression. Just like the corneal reflex, tracking the head marker allows us to track the motion of the head and infer the movement of the eye in the head, but in a way that is less sensitive to the placement of the illuminators; in addition, due to the size of the marker, it provides high spatial resolution. The only downside is that it requires careful focusing of the camera, as both the pupil and the head marker need to be in focus for optimal resolution.

### Image Analysis

#### Region of Interest Detection

During the experiment, all the image frames acquired (3 per trial at a minimum) were stored on disk. All the image processing aimed at extracting the magnitude of OFRs took place offline, after the experiment was over. The first step, as in any image analysis pipeline, was that of identifying regions within the image in which the features of interest are located. Traditionally, the detection of such regions of interest (ROIs) has been handled by feature-based detection algorithms, usually augmented by heuristic rules tailored to the specific problem at hand. For example, the eye region is often isolated using a 3-stage face and eye detection algorithm based on Haar cascade classifiers [41,42]. Alternatively, the high luminance associated with the corneal reflex can be used to locate the eye, specifically targeting the pupil [43]. More recently, the increase in available computational power and the development of deep-learning neural networks have revolutionized image processing, leading to faster and more robust solutions. For object detection tasks, You Only Look Once (YOLO) networks have proven to be very efficient and accurate [44,45]. Furthermore, YOLO networks can detect multiple objects in a single pass through a neural network, making them exceptionally fast [46,47].

**Figure 2.**
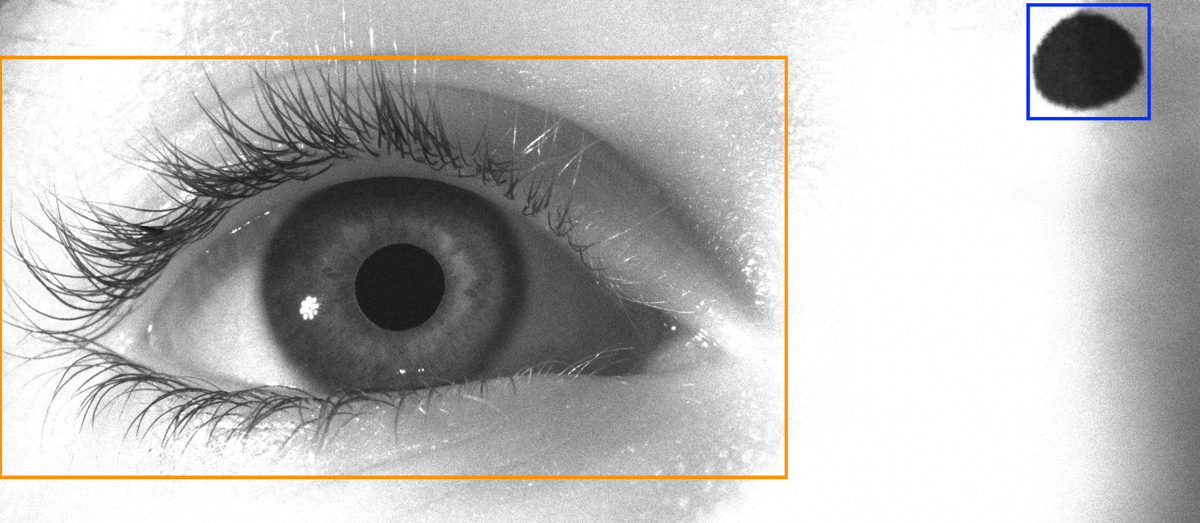
Sample image captured by our eye-tracking system (eye and marker regions of interest identified by the You Only Look Once model are marked with orange and blue rectangles, respectively). The corneal reflections of our infrared illuminators can also be seen to the left of and below the pupil. As the ocular following responses are conjugate, recording a single eye is sufficient [21].

For the first stage of our image processing pipeline, we used the YOLO V8 model to identify the image regions that contain the recorded eye and the head marker (Figure 2). A second YOLO network was then used to identify the pupil area within the eye region detected by the first network. We opted against extracting the pupil and the head marker regions directly because they share similar features. Both models were trained using a custom dataset consisting of 1380 images from 84 subjects. For the first model, we created a dataset in which we used classical computer vision algorithms to annotate the ROIs in each image, followed by manual validation. For the second model, we used traditional algorithms to annotate the pupil region within the eye ROIs extracted in the previous step, again followed by manual validation. These datasets were carefully designed to cover a wide range of eye dimensions, shapes, and (to a smaller extent) head tilts, ensuring that the models exhibited robust performance across diverse subject populations. As no deep-learning solution is infallible, we set a threshold on the confidence score generated by the models for the identification of ROIs to discard images in which the regions could not be identified (eg, when a subject blinked and the pupil was not visible or only partially visible). This ensures the accuracy of the subsequent image processing steps.

#### Head Marker Displacement

As noted above, our goal is to measure the rotation of the eyes within the head over a small time interval (typically 80 milliseconds). To do this, we need to also estimate by how much the head translated in the same period. We do this by measuring the displacement of the head marker across 2 image frames, taken at the beginning and end of the period of interest. Notably, we are not interested in identifying the absolute location of the head marker in each image, only its displacement across images; however, this must be done with subpixel resolution (ideally less than 0.2 pixels).

The solution we have adopted takes the head marker ROIs identified in the 2 images, shrinks them down to a tight rectangle around the marker, then verifies that the rectangles in the 2 images are the same size. This is almost always the case; the exceptions are cases in which there were large movements of the head, and those trials would be discarded anyway. The distance in the image plane of the 2 rectangles gives us the head translation in whole pixels. To find the subpixel fraction of the displacement, we then performed a 2D cross-correlation between the area within the rectangle in one image and the 9 regions with the same area that are within 1 pixel of the rectangle in the other image. This yields a 3 × 3 matrix of cross-correlation values, which we subjected to Fisher *r*-to-*Z* transformation, then bilinearly interpolated to find the subpixel location of the peak. We also used a quadratic interpolation but saw no significant improvement in accuracy. The pixel and subpixel displacements were then added to provide our estimate of head displacement.

#### Pupil Displacement

The solution we adopted for the head marker cannot be used for the pupil because the pupil’s size (and to some extent its shape) changes continuously, even within 80 milliseconds [48,49]. Accordingly, we used an algorithm that extracts the pupil’s center in each image. First, we took the pupil region identified by the YOLO network and shrank it down to a tight rectangle around the pupil. Next, we used a series of filters (ellipses of various sizes and aspect ratios) to locate the pupil center with pixel-level resolution. As the previous step already provided a fairly accurate bounding box for the pupil, only a few filters were needed. If no filter resulted in a good enough match (a very rare occurrence), the trial was discarded. We then fit an anti-aliased annulus to the pupil, restricting our search space to ± 1 pixel around the pixel size identified with the first step. The limiting factor of the accuracy in vivo is the shape of the pupil (which is not always perfectly elliptical). More importantly, its location is not rigidly tied to the direction of the visual axis, introducing a hard limit on the spatial resolution of any pupil-based eye position determination [50-52], making estimates of accuracy based on an artificial eye, found on commercial system specifications, hard to translate in practice. The displacement of the eye (in the plane of the image) was then computed by simply subtracting the coordinates of the center of the ellipses fitted to the pupil in the 2 images. Finally, the displacement of the eye in the head was estimated by subtracting from this pupil displacement the displacement of the head marker computed previously.

## Results

### Overview

To assess our system’s performance, we: (1) evaluated the resolution of our head marker displacement algorithm in artificial and real-use settings, (2) compared the OFRs measured simultaneously with our system and with a scleral search coil in an experienced adult subject, and (3) evaluated the ability of the system to measure OFRs in a clinical setting. As mentioned previously, we consider calibration results obtained with an artificial eye, common in the literature, to be of limited value in practice, and did not pursue that approach.

### Artificial Marker Calibration

We mounted a head marker on a plastic block attached to a manual X-Y micro manipulator rigidly mounted to the end of a table. On the same table, we rigidly mounted our camera approximately 50 cm from the head marker. We then displaced the marker in small increments (by a total distance of up to 180 μm) either horizontally, vertically, or diagonally, and manually triggered the camera to acquire images of the marker at various displacements. Horizontal and vertical series were repeated with either a fine (7.5 μm) or coarse (22.5 μm) increment; only the fine increment was used for the oblique displacements. The displacement of the marker between each of the images and the initial reference image was then extracted with the algorithm described.

**Figure 3.**
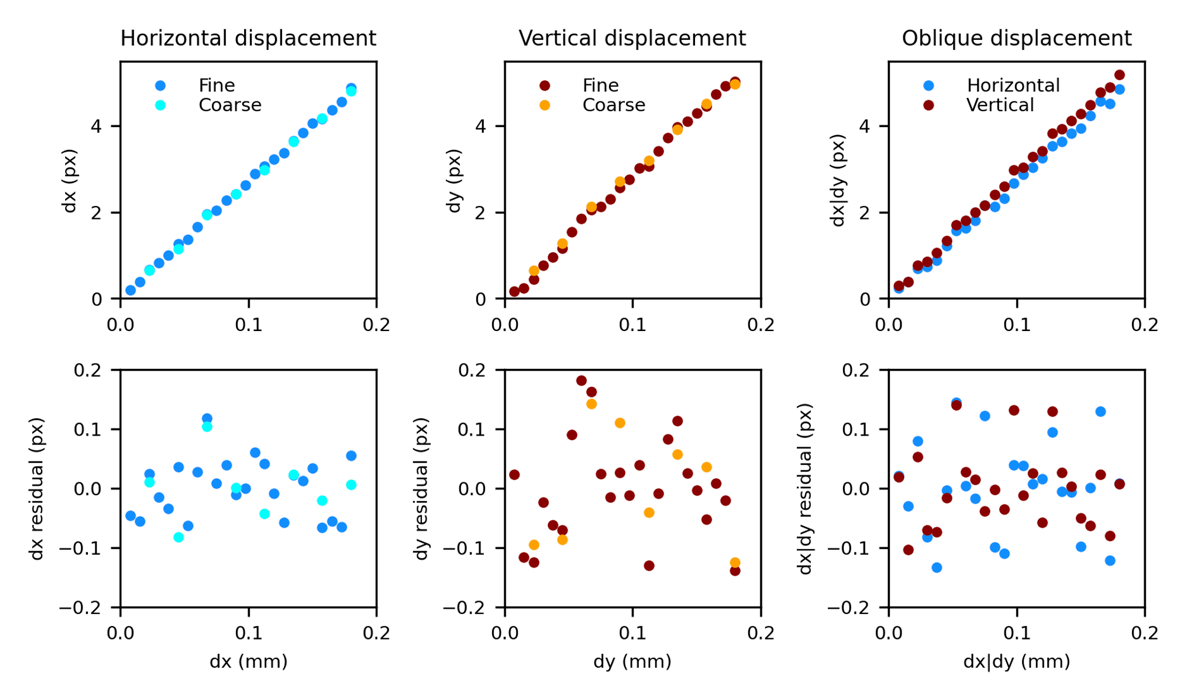
Artificial marker calibration results. Top row: estimated displacement (y-axis, in pixels) and actual displacement (x-axis, in mm) of the marker across all experiments. Bottom row: residuals after linear regression. Px: pixels.

In Figure 3, we plotted the estimated (in pixels) and actual (in mm) displacement of the marker for all experiments, as well as the residuals obtained by regressing out from the estimated displacements a linear prediction based on the data.

The slope of the regression matches what we expected from the geometry of the setup, with a slight difference in slope between horizontal and vertical displacements, likely due to an imperfect alignment of the camera. The standard deviation of the residuals varied between 0.05 pixels in the horizontal direction and 0.08 pixels in the vertical direction (corresponding to 2‐3 μm of displacement). This is better than our desired 10 μm accuracy, although the conditions of this test (ideal lighting, minimal vibrations, accurate focus) are not those of everyday practice.

### Reliability Estimation of Head-Tracking Method in an Adult Population

To estimate the reliability of our head-tracking method under actual recording conditions, we recruited 7 subjects (6 males, 1 female, aged 21‐51 years). All subjects had healthy vision (normal or with slight myopia), did not wear eyeglasses or contact lenses during the experiment, and had no difficulties seeing or fixating their gaze on the fixation target. Note that the goal of this experiment was to evaluate our ability to detect small head movements; therefore, small uncorrected optical deficits were inconsequential. We presented to our subjects moving stimuli known from previous studies to induce strong OFRs: a patch of high-contrast random dots (Figure 1B) that drifted either up or down at 50°/s within a 28° diameter circular aperture. During each trial, we captured 4 frames at specific time points: t0=0 milliseconds, t1=80 milliseconds, t2=100 milliseconds, and t3=180 milliseconds. Given that our system extracts the displacement of the head marker’s position across pairs of frames, if it were perfectly accurate, the sum of the displacements between frames t0 and t1 and between frames t1 and t3 should exactly match the sum of the displacements between frames t0 and t2 and between frames t2 and t3 (in both cases representing the displacement between frames t0 and t3). Differences between these 2 sums represent a measurement error.

We found that, across subjects, the mean (SD) of the head displacement measurement error (computed as described previously) was 0.09 pixels in the horizontal direction and 0.11 pixels in the vertical direction (corresponding to <5 μm of displacement). As expected, this is worse than in the artificial setting described before, but still better than our requirements.

### Comparative Evaluation of the Designed System and Scleral Coils for Measuring OFRs

To evaluate the overall ability of our system to measure OFRs, we then fitted a subject with a scleral search coil and performed an experiment in which we presented a high-contrast random dot stimulus that drifted either up or down at 50°/s within a 28° diameter circular aperture. The subject (male, 51 years old) was highly experienced, having participated in hundreds of OFR recording sessions with scleral eye coils, and was used to the discomfort associated with wearing coils and to suppressing saccades and blinks. We recorded the induced OFRs using both the search coil and our video-based recording system. This was repeated twice; in the first session, we placed the camera on the side of the monitor (ie, a little farther from the subject), while in the second session, we placed it under the monitor (ie, closer to the subject; this was the location of choice for all our subsequent uses of the system).

**Figure 4.**
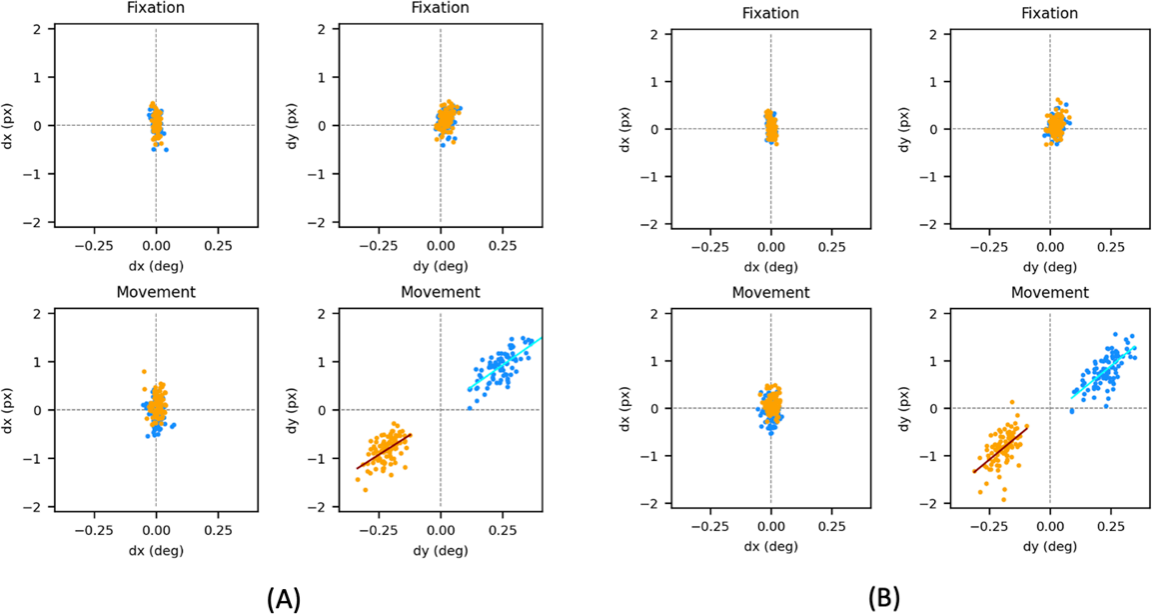
Simultaneous ocular following response recordings with search coils and our eye tracker in a single adult subject. (A) Setup with the camera on the right of the monitor (1 pixel corresponds to approximately 0.29°). (B) Setup with camera under the monitor (1 pixel corresponds to approximately 0.24°). Each dot represents a different trial, color-coded based on the stimulus direction of motion (orange=downward, blue=upward); we plotted the eye-in-head displacement based on coil measurements (in degrees) on the x-axis, while the displacement based on our eye tracker measurements (in pixels) was plotted on the y-axis. Horizontal and vertical eye displacements in the head were plotted separately for the fixation (0‐80 milliseconds, top row) and movement (80‐160 milliseconds, bottom row) epochs. The rationale for selecting these epochs is outlined in the Methods section (Requirements). Deg: degrees; px: pixels.

In Figures 4A and B, we plotted the results of these experiments, showing separately horizontal and vertical deviations measured with the 2 systems (coils on the abscissa in degrees, eye tracker on the ordinate in pixels) during the fixation and movement windows. Using a highly practiced subject with steady fixation has the advantage of evaluating the system under close to ideal conditions. We separately analyzed responses to upward (blue) and downward (orange) drifting stimuli. Converting the pixel displacements extracted by our software into equivalent degrees of visual angle, we found consistent results across the 2 sessions. During the fixation epoch, the variability (SD) of the coil signal ranged between 0.01° and 0.02°, whereas that of the eye-tracking system ranged between 0.03° and 0.05°. During the movement period, along the horizontal direction, the variability of the coil signal was 0.02°, while that of the eye tracker ranged from 0.04° to 0.06°.

During the movement period, the scatter in the vertical direction was considerably larger (SD between 0.04° and 0.06° for the coil, and 0.08° for the eye tracker), but part of that scatter was due to variability in the underlying movement that is being measured (ie, the OFRs).

If we assume that all the shared variability is due to the underlying signal and that the noise of the 2 eye-tracking systems is uncorrelated, we can then assign all the covariance to the OFRs and use the correlation coefficient between the 2 measures to infer the actual variability of each measurement system. This yields a vanishingly small estimate of noise for the coil system and an estimate of the noise introduced by the eye tracker of between 0.06° and 0.07°. Since the coil system cannot have infinite resolution, this indicates that there is some shared variability (possibly due to head movements that were not perfectly compensated for), so these values represent an upper limit estimate of the noise introduced by our video recording system. Its resolution is very close to our desired goal, and it is likely at the limit of what can be obtained with a pupil-tracking system. However, note how much farther away the distributions of values measured for upward versus downward movements along the x-axis are compared to the y-axis, resulting in a d’ measure that is almost twice as large with coils. This highlights the superiority of the coil system in ways that accuracy numbers do not convey.

### Evaluation of System Performance in a Clinical Pediatric Population

As the steady fixation and cooperation typical of a highly experienced subject cannot be expected in a clinical setting, it is also important to evaluate the ability of the system to detect OFRs in a varied clinical population. As one of the goals behind the development of this recording system is to be able to record OFRs in children, we measured OFRs in a cohort of 16 cooperating children (7 males, 9 females, aged 5‐12 years) at the Ophthalmology Department at the Institute for Maternal and Child Health-IRCCS “Burlo Garofolo” (Trieste, Italy). All children underwent a complete ophthalmological and orthoptic examination, with indication of the best corrected visual acuity, the cycloplegic refraction, and the presence of any horizontal and/or vertical manifest strabismus angle with prisms. The inclusion criteria for normal subjects were at least best corrected visual acuity of 20/20 without correction, a cycloplegic refraction between 0.50 and 2.00 diopters, without astigmatism, without anisometropia, and absence of any type of strabismus.

We separately analyzed responses to upward (blue) and downward (orange) drifting stimuli. The displacements of the eye (eye-in-head) during the fixation epoch (frames t1=80 milliseconds versus t0=0 milliseconds) and the movement epoch (frames t2=160 milliseconds versus t1=80 milliseconds) were then computed by subtracting the head marker displacements (head-in-space) from the pupil displacements (eye-in-space). The rationale for selecting these epochs is outlined in the Methods section (Requirements). These measures were all computed in pixels and were then converted to degrees of visual angle based on the geometry of our recording systems, assuming an eye diameter of 22 mm (average for children in this age group) and resulting in a conversion factor of 0.168°/pixel.

**Figure 5.**
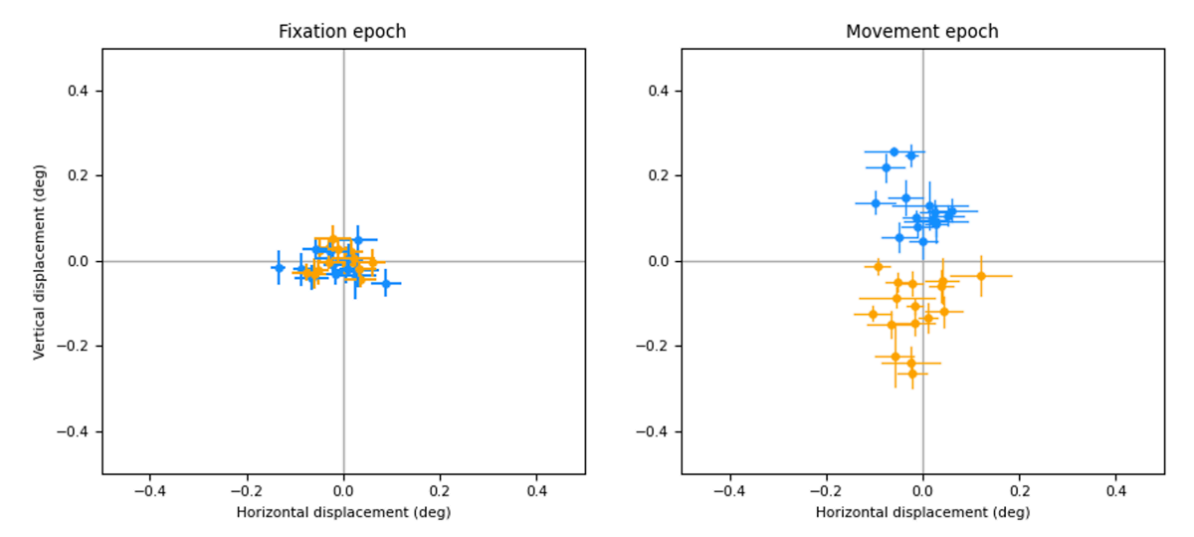
Eye-tracker ocular following response recordings for 16 children in response to stimuli drifting upward (blue) or downward (orange). Average (and ±1 SEM bars) eye displacements recorded in 16 subjects are plotted separately for the fixation (left panel) and movement (right panel) windows. Deg: degrees.

In Figure 5, scatter plots of the average eye displacements for all 16 subjects during the fixation (left) and movement (right) epochs are shown. In the fixation period, there was no significant displacement (unpaired 2-tailed *t* test, *P*>.05) in any of the subjects. In the movement epoch, all subjects showed significant differences (unpaired 2-tailed *t* test, *P*<.05) between vertical displacements induced by vertically drifting stimuli (upward versus downward).

This demonstrates that, with this system, it is possible to measure OFRs in children in a clinical environment, with minimally instructed subjects, during a single, brief recording session lasting less than 3 minutes.

### Pupil Diameter Changes as a Possible Cause of Error in the Measurement

**Figure 6.**
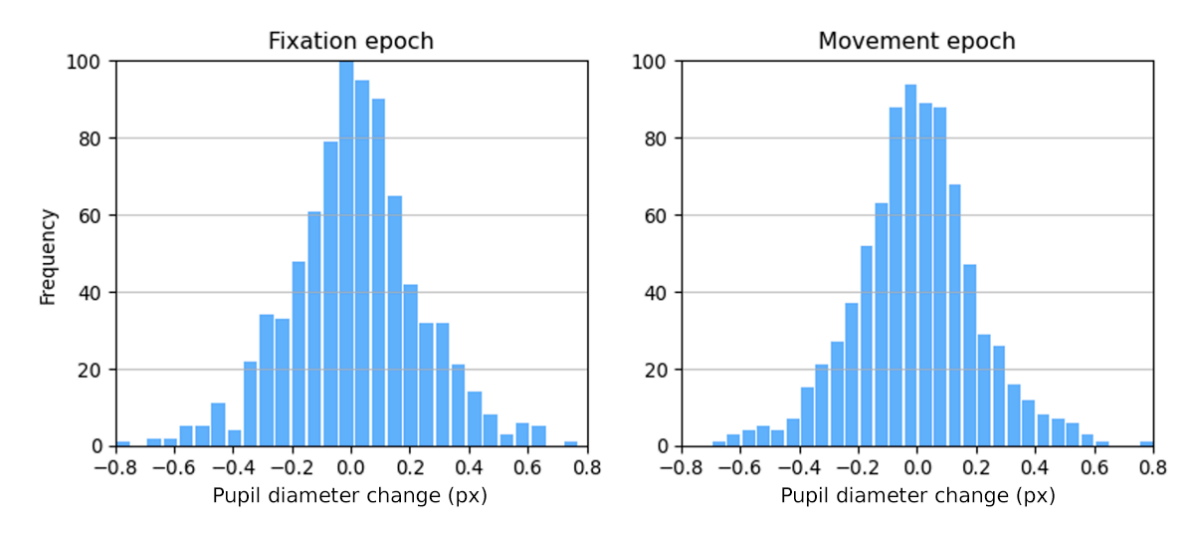
Distribution of pupil diameter changes (expressed in camera pixels) during the fixation (left panel) and movement (right panel) epochs, pooled across 16 subjects.

The data from the previous experiment allowed us to quantify the variation in pupil size that can be expected in OFR experiments. In Figure 6, we report the distribution of pupil diameter changes (expressed in camera pixels) that we observed in the 16 pediatric subjects during the fixation (mean 0.01, SD 0.22) and movement epochs (mean 0.00, SD 0.21). Since the SD of the pupil diameter change is equal to our desired accuracy, changes in pupil diameter cannot simply be assumed to be negligible and ignored.

## Discussion

Eye movements can provide a window through which we can gain insight into brain function and dysfunction [53-55]. This has long been recognized; over the years, several methods for recording eye movements have been developed, leading to numerous scientific and clinical discoveries. Unfortunately, because of the challenges in recording them, the potential diagnostic value of OFRs has only been recognized recently.

Here, we described a system that, thanks to a spatial resolution that approaches that of a magnetic search coil system, allows for the recording of OFRs noninvasively in a pediatric clinical setting. The system could potentially be useful for the identification of stereodeficiencies in young children and nonverbal adults. Due to the sensitivity of OFRs to motion and disparity signals, more widespread recordings in clinical populations will likely lead to additional clinical applications of the proposed solution.

Traditional eye movement recordings methods such as scleral search coils and dual-Purkinje eye trackers offer high precision but are expensive and complex to use. In addition, the former is invasive, while the latter requires strict head stabilization, both undesirable in pediatric populations. Commercial, video-based eye-tracking systems are now in widespread use, despite their often-high cost. However, they lack the desired spatial accuracy [35], thus requiring extensive averaging over long recording sessions, making them impractical for routine clinical use. Our system addresses these problems.

Further improvements in spatial resolution are possible but would not be easy to achieve. As the pupil changes shape and size constantly (Figure 6) and wobbles in the eye as it moves [48-52], significantly higher resolutions based on pupil tracking are probably unachievable. Tracking the motion of the iris would be a natural next step, but partial occlusion from the eyelids introduces hurdles, especially for vertical movements. In any case, increases in the spatial resolution of eye tracking would have to be matched by improved spatial resolution in the detection of head movements, an equally daunting task.

The proposed system, thanks to its demonstrated ability to noninvasively record OFRs in short recording sessions, offers a versatile solution suitable for clinical applications, particularly in a pediatric population, where eye tracking is more challenging. The system could potentially be suitable for diagnostic applications, particularly in pediatric populations, where early detection of visual disorders like stereodeficiencies is critical.

## Abbreviations

IR: infrared
IRCCS: Istituto di Ricovero e Cura a Carattere Scientifico
OFR: ocular following response
ROI: region of interest
TTL: transistor-transistor logic
YOLO: You Only Look Once
